# Editorial: Brief research reports in pharmacogenetics and pharmacogenomics: 2022

**DOI:** 10.3389/fphar.2023.1172265

**Published:** 2023-03-03

**Authors:** Francesco Sessa, Tarek Magdy, Chonlaphat Sukasem, George P. Patrinos

**Affiliations:** ^1^ Department of Medical, Surgical Sciences and Advanced Technologies “G.F. Ingrassia”, University of Catania, Catania, Italy; ^2^ Department of Pathology and Translational Pathobiology, Louisiana State University Health Science Center-Shreveport, Shreveport, LA, United States; ^3^ Feist-Weiller Cancer Center, Louisiana State University Health Science Center-Shreveport, Shreveport, LA, United States; ^4^ Division of Pharmacogenomics and Personalized Medicine, Department of Pathology, Faculty of Medicine Ramathibodi Hospital, Mahidol University Bangkok, Bangkok, Thailand; ^5^ Pharmacogenomics and Precision Medicine, The Preventive Genomics & Family Check-up Services Center, Bumrungrad International Hospital, Bangkok, Thailand; ^6^ Department of Pharmacy, School of Health Sciences, University of Patras, Patras, Greece; ^7^ Department of Genetics and Genomics, College of Medicine and Health Sciences, United Arab Emirates University, Al Ain, United Arab Emirates; ^8^ Zayed Center for Health Sciences, United Arab Emirates University, Al Ain, United Arab Emirates

**Keywords:** pharmacogenetics, pharmacogenomics, genotyping, pharmacogenome, genome-wide association studies

The scientific basis of pharmacogenetics dates back to the 1950s when it was ascertained that drug responses were influenced by genetic characteristics, leading to different responses in patients. Since then, pharmacogenetic studies have gradually grown to reach their zenith today: for example, entering the two main keywords of this Research Topic, “Pharmacogenetics” and “Pharmacogenomics”, in the international database PubMed (retrieved from https://pubmed.ncbi.nlm.nih.gov/), the results demonstrate an exponential growth from 2000 to date. From 1961 to 1999, international publications did not exceed 200 papers/year, achieving almost 1,000 papers/year in 2010 (993 articles), and 1,265 in 2022. This background shows a growing interest of the international scientific community in this research field, taking into account that knowledge of gene-drug interactions may evolve into therapeutic applications. Despite the fact that many steps forward have been made thanks to a multidisciplinary approach with the involvement of tools from different disciplines, such as biochemistry, molecular biology, statistics, and computer science, many questions remain open.

This Research Topic aimed to shed light on recent progress in the Pharmacogenetics and Pharmacogenomics field as well as current and future challenges, aiming to provide a thorough overview of the state of the art in this field, focusing on three milestones: the exploration of the pharmacologically relevant variome in humans and animals from single nucleotide polymorphisms to large structural variations; - method and strategy development and applications of clinical translation in the field; - ethical, legal, economic, and drug developmental aspects of pharmacogenetics/genomics.

The Research Topic comprises 4 articles to which 28 authors have contributed, while several others that have been submitted did not pass the stringent quality criteria that we have set.

Alshabeeb and coworkers (Alshabeeb et al.) focused their research article on idiosyncratic drug-induced liver injury (DILI), a serious uncommon disease, that could be developed after the intake of different drugs such as the antimicrobials flucloxacillin and co-amoxiclav. Their hypothesis is based on previously published papers that reported an association between DILI and the solute carrier organic anion transporter 1B1 (SLCO1B1), describing that it could be considered a risk factor for liver injury induced by rifampin and methimazole. Particularly, the authors investigated the association of rs4149056 (SLCO1B1*5, c. T521C) variant with flucloxacillin- and co-amoxiclav–induced liver injury. Based on the results of this study, the examined allele SLCO1B1*5 may not be considered a risk factor for flucloxacillin DILI or co-amoxiclav DILI, as previously suggested. The authors suggested that future studies should be performed in order to clarify the role of a different allele (SLCO1B1*1B) that could be involved in DILI; moreover, they suggested focusing on another family member gene (SLCO1B3) that could be analyzed in order to drive the choice of antimicrobial drugs.

Blazy and coworkers (Blazy et al.) performed a retrospective chart review of patients who were pharmacogenetic typed, in order to guide psychotropic use. The authors focused on the results of 205 patients enrolled in their study, comparing the results for CYP2D6 and CYP2C19 obtained using the commercial pharmacogenomic phenotype test to those obtained from Clinical Pharmacogenetics Implementation Consortium (CPIC) guidelines. The results of this study demonstrated that the prevalence of conflicting phenotype assignment was about 30% for both tests (28.8% for *CYP2D6* and 32.2% for *CYP2C19* genotypes). Based on these results, the authors highlight the importance of the phenotypic assignment, in particular for antidepressants drug, considering the implications for dose adjustments based on CYP2D6 or CYP2C19 metabolizing phenotype.

Kee and coworkers (Kee et al.) analyzed the importance of pharmacogenomic phenotype for omeprazole, a drug used to manage gastroesophageal reflux disease (GERD). Particularly, it has been described that the CYP2C19*17 (rs12248560) allele and the recently described *CYP2C:TG* haplotype (rs11188059 and rs2860840) may be associated with increased enzymatic activity, reducing the omeprazole exposure. They performed an observational study recruiting not respond patients to omeprazole. Of a total of 55 cases, 19 (34.5%) were CYP2C19*17 heterozygotes and two (3.6%) were CYP2C19*17 homozygotes. Based on the results of TG haplotypes, 7 were (12.7%) *CYP2C*:TG homozygotes, and 16 (29%) *CYP2C*:TG heterozygotes. Applying gastroscopy and 24-h esophageal pH/impedance tests, the authors determined that there was objective evidence of GERD in a subgroup of 39 (71%) cases, concluding that omeprazole treatment failure is associated with *CYP2C*:TG/TG, but not CYP2C19*17.

Finally, Resino and coworkers (Resino et al.) focused their research activities on immunodeficiency virus (HIV)-infected patients in treatment with antiretroviral drugs who had a lack of recovery of CD4^+^ T-cells (CD4^+^ recovery). For this reason, they analyzed the association between single nucleotide polymorphisms (SNPs) underlying vitamin D metabolism and the CD4^+^ recovery in naïve HIV-infected patients who started antiretroviral therapy with low baseline CD4^+^. Particularly they focused on two genomic variants: *DBP* rs7041 (AA *versus* CC/AC) and *DHCR7* rs3829251 (AA *versus* GG/AG). Based on their results, CD4^+^ recovery was higher in patients carrying *DBP* rs7041 AA genotype as well as *DHCR7* rs3829251 AA genotype, concluding that both polymorphisms were associated with CD4^+^ recovery in HIV-infected patients.

In conclusion, the published articles in this Research Topic covered interesting and various aspects of the theme, proposing new findings that will help to gain ground in the development of Pharmacogenetics and Pharmacogenomics and their implementation in clinical practice. Moreover, it is desirable that well-set-out studies will be carried out in order to improve knowledge in this challenging field.

